# Delta-secretase cleavage of Tau mediates its pathology and propagation in Alzheimer’s disease

**DOI:** 10.1038/s12276-020-00494-7

**Published:** 2020-08-28

**Authors:** Seong Su Kang, Eun Hee Ahn, Keqiang Ye

**Affiliations:** Department of Pathology and Laboratory Medicine, Emory University School of Medicine, Atlanta, GA 30322 USA

**Keywords:** Neurodegeneration, Alzheimer's disease

## Abstract

Alzheimer’s disease (AD) is a progressive neurodegenerative disease with age as a major risk factor. AD is the most common dementia with abnormal structures, including extracellular senile plaques and intraneuronal neurofibrillary tangles, as key neuropathologic hallmarks. The early feature of AD pathology is degeneration of the locus coeruleus (LC), which is the main source of norepinephrine (NE) supplying various cortical and subcortical areas that are affected in AD. The spread of Tau deposits is first initiated in the LC and is transported in a stepwise manner from the entorhinal cortex to the hippocampus and then to associative regions of the neocortex as the disease progresses. Most recently, we reported that the NE metabolite DOPEGAL activates delta-secretase (AEP, asparagine endopeptidase) and triggers pathological Tau aggregation in the LC, providing molecular insight into why LC neurons are selectively vulnerable to developing early Tau pathology and degenerating later in the disease and how δ-secretase mediates the spread of Tau pathology to the rest of the brain. This review summarizes our current understanding of the crucial role of δ-secretase in driving and spreading AD pathologies by cleaving multiple critical players, including APP and Tau, supporting that blockade of δ-secretase may provide an innovative disease-modifying therapeutic strategy for treating AD.

## Alzheimer’s disease: Enzyme’s role in disease progression

The identification of an enzyme that plays a critical role in the progression of Alzheimer’s disease (AD) could lead to novel therapeutic interventions. In the earliest stage of AD, the build-up of Tau protein aggregates causes degeneration of a site in the brainstem. These abnormal Tau accumulations then spread to other parts of the brain. Recent research suggests that an enzyme called delta-secretase cleaves Tau and other key molecules, making Tau more prone to forming aggregates and thus facilitating disease progression. Keqiang Ye and co-workers at Emory University School of Medicine in Atlanta, USA, reviewed current understanding of the role of delta-secretase in AD pathology. Studies show that delta-secretase expression levels are high in aged mice and AD brains. Inhibiting delta-secretase could therefore limit neurodegeneration and alleviate cognitive deficits in patients.

## Introduction

Alzheimer’s disease (AD) is an age-mediated progressive neurodegenerative disorder and is a type of dementia that causes problems with memory, thinking, and behavior. Symptoms usually develop slowly and worsen over time, becoming severe enough to interfere with daily tasks. The pathology of AD is characterized by the accumulation of β-amyloid peptide (Aβ) within the brain along with hyperphosphorylated and cleaved forms of the microtubule-associated protein Tau. In addition, AD pathology also includes extensive neuronal loss, accompanied by chronic neuroinflammation. It is known that metabolic dysfunction of amyloid β precursor protein (APP) and abnormal Tau protein phosphorylation lead to the formation of neuritic plaques and neurofibrillary tangles (NFT), respectively^1^. These events finally drive the clinical expression of dementia. Genetic, biochemical, and behavioral studies suggest that pathologic generation of the neurotoxic Aβ peptide from sequential APP proteolysis is the crucial step in the development of AD, and APP is metabolized in a rapid and highly complex fashion by a series of sequential secretases, including β-secretases (BACE1), γ-secretase (γ-SC) and the ADAM family as α-secretases^2^. On the other hand, the proteolysis of Tau also plays an important role in both Tau aggregation and neurodegeneration. Tau is a substrate of several endogenous proteases, including caspases, thrombin, calpain, and AEP^3–5^. Cleavage of Tau cripples its physiological microtubule-associated functions in axons and leads to aberrant aggregation and pathological neurotoxicity^6^. In AD, the burden of Tau aggregates correlates closely with neuronal cell death and cognitive decline, and Tau aggregates alone cause neurodegeneration in other tauopathies^7,8^. Remarkably, Tau PET imaging studies also show that Tau inclusion correlates with clinical symptoms and neurodegeneration in human AD^9^. Hence, Tau plays a key role in neurodegeneration.

## Tau pathology in Alzheimer’s disease

The gradual deposition of hyperphosphorylated Tau proteins within select neuronal types in specific nuclei or areas is central to the disease process. The staging of AD-related pathology was performed on unconventionally thick sections (100 μm) using a silver technique and reflected the progress of the disease process based chiefly on the topographic expansion of the lesions^10^. The pattern of spread of the Tau aggregation pathology in the human brain is highly characteristic and stereotyped. In the cortex, it begins in layer II of the entorhinal cortex. The pathology spreads via the perforant pathway to the hippocampus. Protrusions from the hippocampus return to layer IV of the entorhinal cortex and to other limbic structures. Subsequently, the pathology spreads into the isocortex, initially into the temporal and parietal lobes, and eventually into the frontal and occipital neocortex. This pattern of progression and spread forms the basis of the 6-stage Braak staging system for neurofibrillary degeneration in AD^11^. Braak has also provided a corresponding staging system for β-amyloid deposition, with three levels of amyloid deposits: no deposits and three levels with increasing amyloid (stages A–C), depicting the tempo-spatial distribution of senile plaques and NFT pathology in AD progression^12^.

## Tau pathology in the locus coeruleus

NFT comprise a dense whorl of fibrils occupying the entire perinuclear cytoplasm of cortical pyramidal cells and other large neurons in the brainstem (nucleus basalis of Meynert and locus coeruleus (LC)). These fibrils are termed paired helical filaments (PHFs), which are composed of Tau, and the core of its constituent filaments is made of truncates from the repeat domain of Tau. The degeneration of the brainstem LC, the major norepinephrine (NE)-producing nucleus in the brain, is one of the hallmarks in AD^13^. Because of the importance of the LC for the regulation of attention, arousal, and mood, LC degeneration has been suggested to be responsible for certain neuropsychiatric abnormalities that are common in AD, such as anxiety, depression, and sleep disorders^14,15^. Hyperphosphorylated Tau, a “pretangle” form of the protein that is prone to aggregation, can be detected in the LC before elsewhere in the brain, sometimes during the first few decades of life^16–19^. The “seeding” hypothesis postulates that misfolded proteins originate in one population of neurons and then spread to interconnected brain regions via a prion-like process of corruptive templating^20^. Because hyperphosphorylated Tau is first detected in the LC and the LC sends dense projections to other vulnerable brain regions that display early Tau pathology, it has been suggested that the LC might be one of the origins (perhaps along with other affected brainstem nuclei that project to the trans-entorhinal cortex such as the dorsal raphe) of Tau neuropathology in AD^21^. These proteolytic truncated fragments catalyze the conversion of normal soluble Tau into aggregated oligomeric and fibrillar Tau, which, in turn, can spread to neighboring neurons. Tau aggregation is not a late-life process, and the onset of Braak stage 1 peaks in people in their late 40 s or early 50 s. Tau aggregation pathology at Braak stage 1 or beyond affects 50% of the population over the age of 45. The initiation of Tau aggregation requires its binding to a nonspecific substrate to expose a high-affinity tau–tau binding domain, and it is self-propagating thereafter. The initiating substrate complex is most likely formed as a consequence of a progressive loss of endosomal-lysosomal processing of neuronal proteins, particularly of membrane proteins from the mitochondria^12^. Thus, early pathology in the LC could initiate a vicious cycle in which aberrant Tau induces LC hyperactivity, thereby promoting its own spread to interconnected brain regions via trans-synaptic propagation and facilitating the progression of disease^22^.

In the central nervous system (CNS), the catecholamines dopamine, norepinephrine, and epinephrine are intraneuronally metabolized to their respective aldehyde metabolites by monoamine oxidase (MAO). Dopamine is deaminated to 3,4-dihydroxyphenylacetaldehyde (DOPAL), and both norepinephrine and epinephrine are deaminated to form 3,4-dihydroxyphenylglycolaldehyde (DOPEGAL). Mounting evidence supports that these catecholamine-derived aldehydes may in fact be neurotoxins, and their intraneuronal accumulation has been theorized as one mechanism that may be involved in cell death associated with neurodegenerative conditions, including Parkinson’s disease (PD) and AD^23^. Experimental ablation of the LC exacerbates, while increasing NE abates, AD-like neuropathology and cognitive deficits in animal models of the disease^24^. There is an increasing body of evidence demonstrating the neurotoxic properties of the catecholamine-derived aldehydes DOPAL and DOPEGAL by various cytotoxic mechanisms, including the generation of free radicals and initiation of apoptosis^25,26^. These aldehydes are generated due to the passive leakage of catecholamines into the cytoplasm from stored vesicular monoamine transporter 2 (VMAT2), which are subsequently oxidized by MAOs in catecholamine neurons. The concentration of DOPEGAL in normal postmortem LC is estimated to be 1.4 μM, a level ~50% of that of 3-methoxy-4-hydroxyphenylglycol (MHPG), a major metabolite of norepinephrine and epinephrine^27^. Normal postmortem human brain SN levels of DOPAL are estimated to be 2.3 μM, a level ~25% higher than that of homovanillic acid (HVA), a major dopamine metabolite. In addition to in vitro cytotoxicities by DOPAL and DOPEGAL, in vivo cytotoxicity of DOPAL has been reported in neurons and glia in the SN and ventral tegmental area (VTA)^23^. The in vivo toxicity of DOPEGAL has also been demonstrated in rat rostral ventrolateral medulla (RVLM)^28^. Various mechanisms have been suggested to explain the observed cytotoxicity of DOPAL and DOPEGAL. These include protein adduction, isoquinoline formation, and free radical generation^29^. For instance, DOPAL covalently modifies α-synuclein and triggers its oligomerization, leading to synapse physiology impairment in PD^30,31^. Recently, we reported that DOPAL stimulates the interaction between α-synuclein and TrkB and blocks its neurotrophic signaling. Accordingly, the MAO-B inhibitor rasagiline blocks DOPAL production, disrupts the α-synuclein/TrkB complex and rescues TrkB neurotrophic signaling, preventing α-synuclein-induced dopaminergic neuronal death and restoring motor functions. Our findings demonstrate a noble pathological role of α-synuclein in antagonizing neurotrophic signaling, providing a molecular mechanism that accounts for its neurotoxicity in PD^32^.

## Delta-secretase drives AD pathologies by cleaving both APP and Tau

**Fig. 1:**
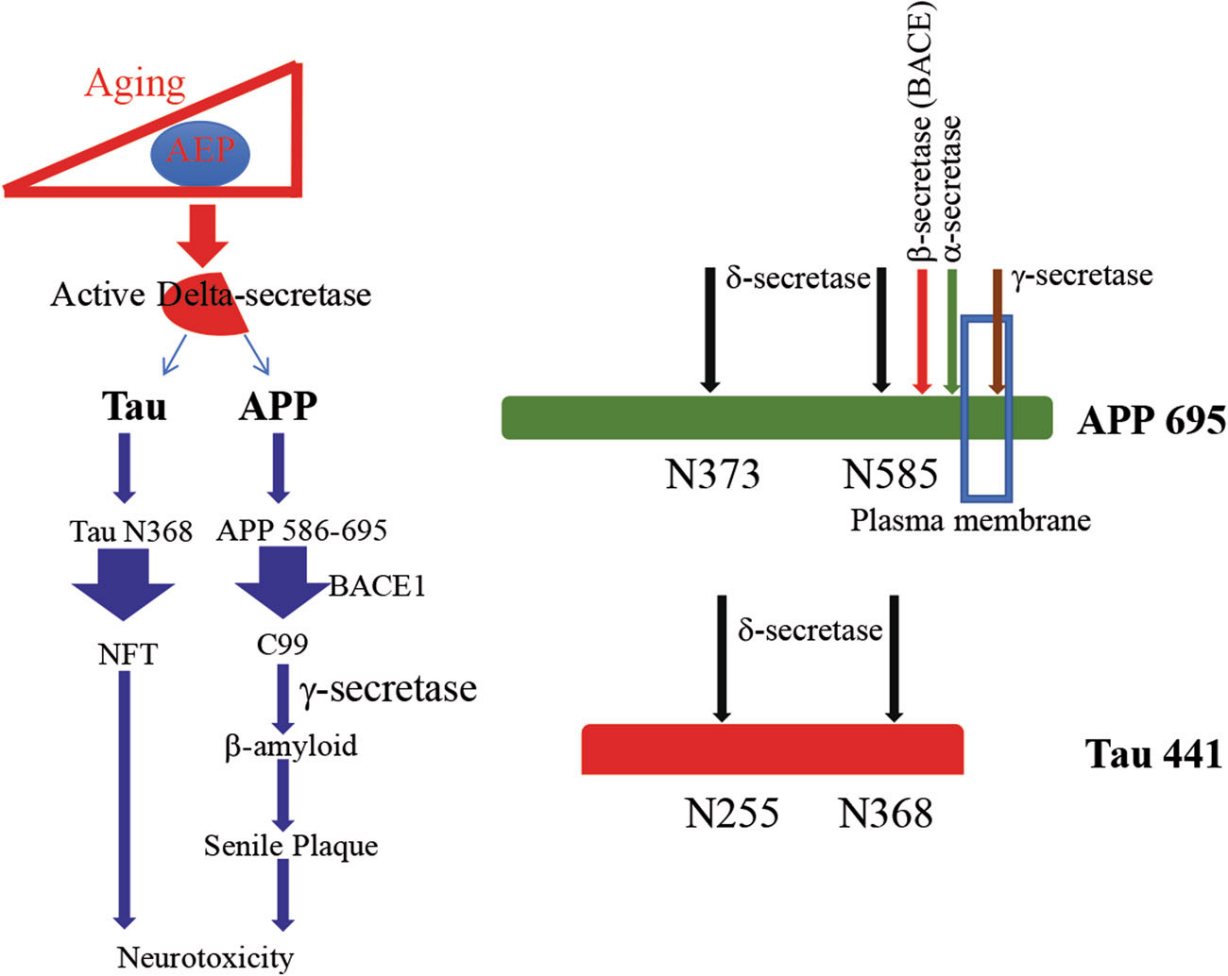
Active δ-secretase triggers AD pathologies by cleaving both Tau and APP. The schematic model demonstrates that AEP (δ-secretase) is activated in an age-dependent manner and cleaves APP at N585 and Tau at N368, which triggers AD pathology.

**Fig. 2:**
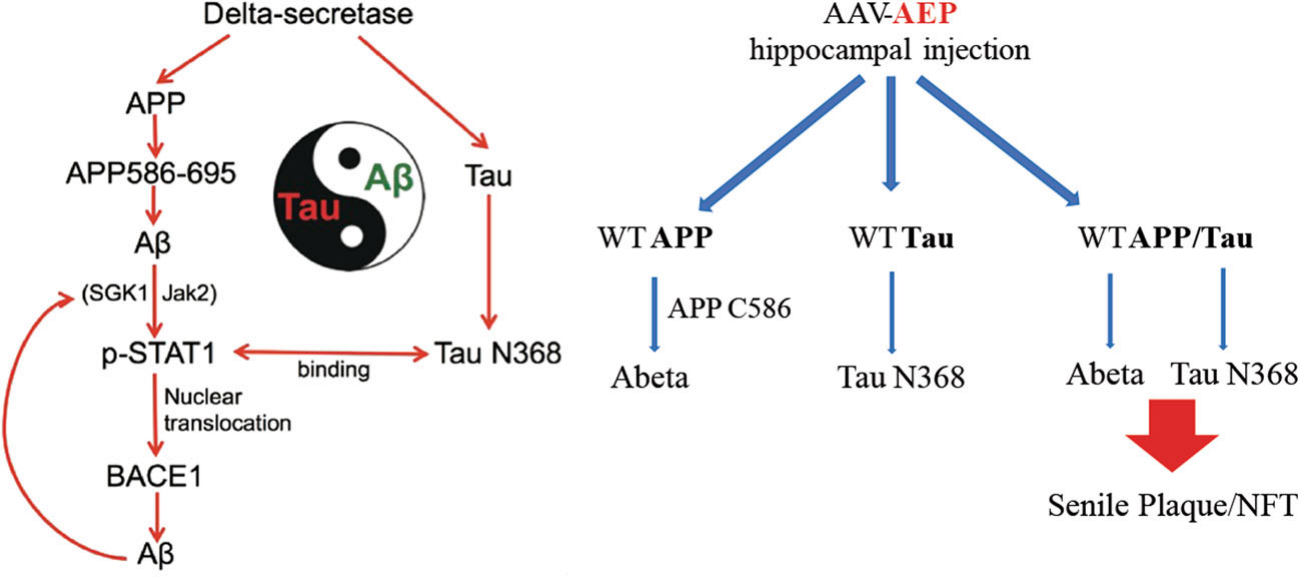
Schematic model of δ-secretase-generated APP and Tau fragments on BACE1 expression. δ-Secretase cleaves APP, generating an APP (586–695) fragment, which is further cleaved by β-secretase and γ-secretase to produce Aβ. Aβ induces the phosphorylation of SGK1 and JAK2, which subsequently activates STAT1. On the other hand, δ-secretase cleaves Tau and generates a Tau (1–368) fragment, which binds active STAT1, promoting its nuclear translocation. Activated STAT1 enhances the transcription of BACE1 and the production of Aβ (left). Right. An in vivo model shows that hippocampal injection of δ-secretase into wild-type human APP/Tau double transgenic mice evidently accelerates large senile plaques and NFT formation.

Mammalian asparaginyl endopeptidase (AEP; also called legumain) is a lysosomal cysteine protease that cleaves after asparagine residues. AEP is most abundant in the kidney, spleen, liver, placenta, testis, and thymus. AEP activation is autocatalytic and requires sequential removal of C- and N-terminal propeptides at different pH thresholds^33^. Disruption of AEP leads to late endosome and lysosome augmentation and dislocation from the apical region of kidney-proximal tubule cells and abnormal lysosomes contained in electron-dense and/or membranous materials^34^. Recently, we reported that AEP is involved in neuronal cell death by degrading the DNase inhibitor SET during excito-neurotoxicity. Ischemia or kainic acid induces acidosis in the brain and activates AEP, which subsequently cleaves SET at the N175 residue and abolishes its DNase inhibitory activity, leading to neuronal cell death^35^. AEP also cleaves TDP-43 at multiple sites after asparagine residues and is implicated in frontotemporal lobar degeneration (FTLD)^36^. Physiologically, AEP is inhibited in lysosomes by cystatin C, an Aβ plaque colocalized protein^37^. Cystatin C binds soluble Aβ and inhibits its oligomerization, preventing neurodegeneration in AD^38^. Recently, we reported that AEP cleaves both APP and Tau in the brain in an age-dependent manner. Both APP and Tau are robustly fragmented by AEP in human AD brains. Hence, AEP possesses delta-secretase (δ-secretase) activity. Interestingly, δ-secretase expression levels and activities are increased in aged mice and AD brains compared to young mice or control human brains. Notably, δ-secretase cleaves APP on the extracellular domain at both N373 and N585 residues, promoting Aβ production, which is due to alleviation of the steric hindrance to allow BACE1 to cleave the resultant C-terminal APP (586-695) more efficiently. Knockout of δ-secretase from 5xFAD substantially diminishes Aβ generation and senile plaque deposits, rescuing cognitive deficits^39^. On the other hand, δ-secretase cuts Tau at residues N255 and N368, respectively, promoting Tau hyperphosphorylation and aggregation and neurotoxicities. Deletion of δ-secretase from Tau P301S mice strongly decreases NFT pathology and restores cognitive defects^5^ (Fig. 1). To define the pathological activities of various APP and Tau fragments resulting from δ-secretase cleavage, we injected the hippocampus of wild-type mice with AAV expressing truncates separately or in combination and found that fragments of δ-secretase cleavage, APP (586-695) and Tau (1-368), additively drive AD pathogenesis and cognitive dysfunction. Tau (1-368) strongly augments BACE1 expression and Aβ generation in the presence of APP. Notably, a portion of Tau N368 preferentially resides in the nucleus. Consequently, the Tau (1-368) fragment is more robust than Tau FL (full-length) in binding to active STAT1, a BACE1 transcription factor, and promotes its nuclear translocation, upregulating BACE1 and Aβ production. Thus, we show that Tau may not only be a downstream effector of Aβ in the amyloid cascade hypothesis but also act as a driving force for Aβ when cleaved by δ-secretase^40^. Clearly, Tau N368 preferentially resides in the nucleus compared to Tau FL, accounting for its stimulatory effect in triggering STAT1 nuclear translocation and consequent BACE1 and Aβ upregulation. Most recently, we observed that injection of δ-secretase into the brain of human wild-type APP/Tau double transgenic mice evidently accelerates enormous senile plaques and NFT in both sexes, associated with prominent synaptic defects and cognitive deficits. Hence, δ-secretase drives AD pathogenesis independent of any patient-derived mutation (Wu Z, 2020, unpublished data) (Fig. 2). Consequently, the small molecule inhibitor compound #11 of δ-secretase blocks AD pathology and rescues cognitive deficits^41^.

To delineate how δ-secretase is molecularly regulated, we found that serine-arginine protein kinase 2 (SRPK2), a cell cycle-activated kinase that is agitated in AD brains and mediates neuronal cell death^42^, selectively phosphorylates the S226 residue on δ-secretase and escalates its protease activities. Notably, this phosphorylation elicits δ-secretase cytoplasmic translocation from lysosomes. In addition, δ-secretase is highly phosphorylated in human AD brains and is tightly correlated with SRPK2 activity. Overexpression of a phosphorylation mimetic (S226D) in young 3×Tg mice strongly promotes APP and Tau fragmentation and facilitates amyloid plaque deposits and NFT formation, resulting in cognitive impairment. Conversely, viral injection of the nonphosphorylatable mutant (S226A) into 5XFAD mice decreases APP and Tau proteolytic cleavage, attenuates AD pathologies, and reverses cognitive defects. Hence, δ-secretase phosphorylation by SRPK2 plays a critical role in aggravating AD pathogenesis^43^. SRPK2 plays an important role in pre-mRNA splicing by phosphorylating SR-splicing factors. It is well known that dysregulation of Tau exon 10 pre-mRNA splicing causes pathological imbalances in 3R- and 4R-Tau, leading to neurodegeneration. Interestingly, we show that δ-secretase cleaves SRPK2 at N342 and increases its nuclear translocation as well as kinase activity, augmenting exon 10 inclusion. Conversely, the uncleavable SRPK2 N342A mutant increases exon 10 exclusion, improves synaptic functions, and prevents spatial memory deficits in Tau intronic mutant FTDP-17 transgenic mice. On the other hand, truncated SRPK2 increases 4R-Tau isoforms and accelerates cognitive decline in hTau mice. Hence, δ-secretase also mediates Tau-splicing imbalance in tauopathies by cleaving SRPK2^44^.

**Fig. 3:**
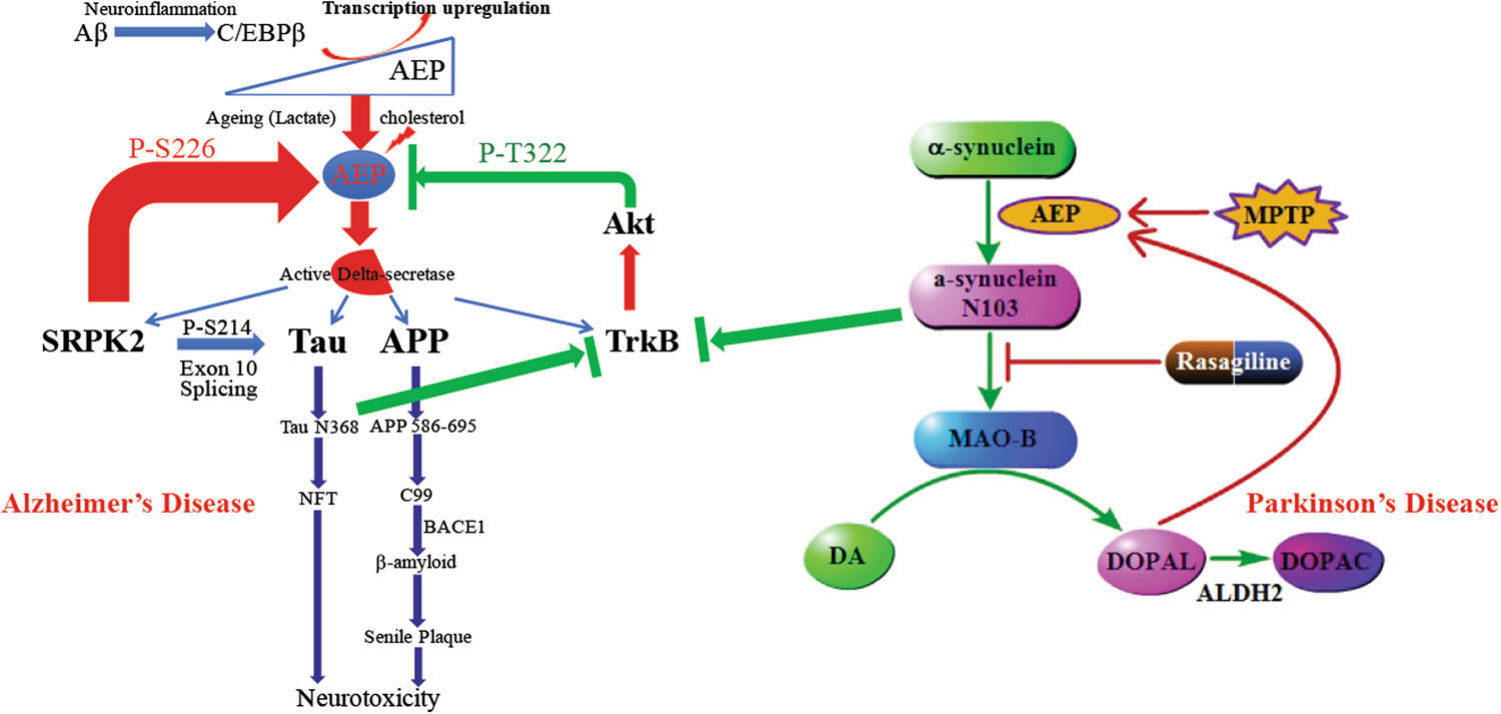
Active δ-secretase is the trigger for both AD and PD. Schematic model of the C/EBPβ/AEP pathway mediating AD pathology. δ-Secretase cleaves numerous substrates, including SRPK2, APP, Tau, and TrkB receptors. SRPK2 phosphorylates δ-secretase on the S226 residue and activates its protease activities. BDNF/TrkB signaling inhibits δ-secretase via Akt phosphorylation on T322, promoting neuronal survival (left panel). A schematic model shows the role δ-secretase in α‐synuclein-induced PD pathology. δ-Secretase‐cleaved α-synuclein N103 triggers MAO‐B activation that feeds forward to further activate δ-secretase by the DA metabolite DOPAL (right panel).

BDNF (brain-derived neurotrophic factor), an essential trophic factor implicated in synaptic plasticity and neuronal survival by binding to its cognate receptor TrkB, is reduced in AD^45^. Interestingly, we found that Akt, a downstream effector of BDNF/TrkB signaling, phosphorylates δ-secretase at residue T322 upon BDNF treatment and triggers its lysosomal translocation and inactivation. When BDNF levels are reduced in neurodegenerative diseases, δ-secretase T322 phosphorylation is attenuated. Consequently, δ-secretase is activated and translocates into the cytoplasm, where it cleaves both Tau and α-synuclein^46^. The association of BDNF deficiency with Tau pathology in AD is well documented. Accordingly, BDNF deprivation triggers Tau proteolytic cleavage by activating δ-secretase, and the resultant Tau N368 fragment binds TrkB receptors and blocks neurotrophic signals, inducing neuronal cell death^47^ (Fig 3, left panel). It is worth noting that the neurotransmitter NE also crosstalks with the TrkB neurotrophic pathway. While increasing NE transmission partially ameliorates neuroinflammation, Aβ load, and cognitive impairment^48,49^, the underlying molecular mechanism accounting for the neuroprotective role of NE in AD remains unclear. Previously, we reported that NE protects primary cortical and LC neurons from Aβ toxicity in a dose-dependent manner. The neuroprotective effects of NE are fully prevented by the Trk receptor antagonist K252a but only partially attenuated by adrenergic receptor antagonists and not mimicked by adrenergic agonists. Therefore, NE activates TrkB receptors and protects against Aβ toxicity, at least in part, via adrenergic receptor-independent mechanisms, and displays implications for the consequences of LC degeneration in AD^24^.

To investigate how aging contributes to δ-secretase escalation, we tested a panel of age-dependent brain expressed transcription factors and found that a CCAAT-enhancer-binding protein (C/EBPβ), an inflammation-regulated transcription factor, acts as a key age-dependent effector elevating both δ-secretase and inflammatory cytokine expression in mediating pathogenesis in AD mouse models. Strikingly, C/EBPβ regulates δ-secretase (gene name *LGMN*) mRNA transcription and protein levels in an age-dependent manner^50^. As expected, the C/EBPβ/δ-secretase axis is activated in an age-dependent manner in different brain regions of the 3×Tg AD mouse model, elevating δ-secretase-truncated APP and Tau proteolytic fragments and promoting senile plaques and NFT formation in the brain, associated with gradual neuronal loss and chronic neuroinflammation. Depletion of C/EBPβ from 3×Tg mice represses APP, Tau, and δ-secretase expression, which subsequently inhibits APP and Tau cleavage, leading to mitigation of AD pathologies. Knockout of δ-secretase from 3×Tg mice strongly blunts AD pathogenesis. Thus, the spatiotemporal activation of the C/EBPβ/δ-secretase axis regulates AD pathogenesis^51^. To dissect the molecular networks between BDNF/TrkB and the C/EBPβ/δ-secretase axis, we withdrew BDNF from primary neuronal cultures and found that deprivation of BDNF/TrkB increased inflammatory cytokines and activated the JAK2/STAT3 pathway, resulting in the upregulation of transcription factor C/EBPβ. This, in turn, increases the expression of δ-secretase, leading to both APP and Tau fragmentation by δ-secretase and neuronal loss. Importantly, a reduction in BDNF/TrkB neurotrophic signaling is inversely coupled with an increase in JAK2/STAT3, C/EBPβ, and δ-secretase escalation in human AD brains^52^. Therefore, these findings provide a mechanistic link between BDNF/TrkB reduction, C/EBPβ upregulation, δ-secretase activity, and Aβ and Tau alterations in the brain. Hence, these discoveries support that δ-secretase is a novel and key age-regulated protease that cleaves both APP and Tau, contributing to AD onset and progression.

## Oxidative stress activates the C/EBPβ/δ-secretase pathway in AD

Oxidative stress, implicated as a major factor in neurodegenerative diseases^53^, affects the functions of aldehyde dehydrogenase (ALDH), a key enzyme involved in aldehyde metabolism. The formation of DOPAL and DOPEGAL aldehydes involves the generation of H_2_O_2_, which inhibits ALDH activity^54^ and, in the presence of ferrous iron, can give rise to hydroxyl radicals that dramatically affect cell viability by initiating lipid peroxidation. Levels of 4-HNE and acrolein, highly cytotoxic lipid peroxidation markers, are elevated in the AD brain^55^, and increased 4-HNE-protein adducts are found in nigral neurons of the PD brain. Oxidative stress damages mitochondria and other organelles, enhances aggregation of α-synuclein^56^, and increases demand on proteosomal and lysosomal degradative systems, further escalating oxidative stress. Dopamine undergoes both enzymatic (via MAO-B) and nonenzymatic oxidation, generating hydrogen peroxide and highly reactive oxygen radicals. Dopamine metabolism also leads to the formation of neuromelanin, which binds reactive metals that can further catalyze the production of free radicals through the Fenton reaction. Consistent with a major role of oxidative stress in PD, PD patients manifest high levels of lipid and protein oxidation as well as depletion of antioxidants such as reduced glutathione (GSH)^57^. Recently, we reported that δ-secretase is activated by DOPAL, a highly toxic and oxidative dopamine metabolite, in the substantia nigra^58^. Active δ-secretase subsequently cleaves α-synuclein at N103 and promotes its aggregation and dopaminergic neuronal loss, leading to motor dysfunction in animal models of PD^59^ (Fig. 3, right panel). Analogous to toxic DA metabolites killing substantia nigra pars compacta (SNpc) neurons in patients with PD, the noradrenergic phenotype of LC neurons itself may contribute to the vulnerability of these cells in AD. NE is converted into DOPEGAL by MAO-A during the normal life cycle of catecholamine production and transmission but is increased in the degenerating LC neurons of patients with AD^60,61^. Injection of DOPEGAL into rodent brains elicits adrenergic neuronal loss^28^, and DOPEGAL toxicity is likely due to the generation of free radicals and activation of mitochondrial permeability transition^25^. The activation of δ-secretase by DOPAL suggests that it might also be activated by DOPEGAL, thus triggering a cascade of events leading to Tau cleavage, hyperphosphorylation, aggregation, and neurotoxicity in LC neurons.

Nuclear erythroid 2-related factor 2 (Nrf2), a redox-sensitive transcription factor, is involved in the regulation of the antioxidant response element (ARE)-mediated expression of phase II detoxifying antioxidant enzymes^62^. In response to oxidative stress, Nrf2 is activated and forms heterodimers with other bZIP proteins, binds to cis-acting element(s) (AREs) in the promoters of target genes, inducing transcriptional responses. Active Nrf2 triggers an immediate induction of C/EBPβ. The C/EBPβ promoter associates with the Nrf2 transcription factor via the ARE binding site during adipogenesis and upregulates its expression^63^. Notably, C/EBPβ is involved in the regulation of proinflammatory gene expression in glial activation and plays a key role in the induction of neurotoxic effects by activated microglia^64^. Although C/EBPβ increases the expression of a wide variety of target genes that regulate numerous metabolic processes, C/EBPβ binding sites are particularly found in regulatory sequences of genes that are associated with the inflammatory response^65^ or the ER stress pathway^66^. When the transactivation domain of C/EBPβ becomes phosphorylated by inflammatory stimuli, transcription of the C/EBPβ gene increases, which subsequently elevates the expression of various proinflammatory genes, including IL-6^67^. MAO-A generates H_2_O_2_ and DOPEGAL when it metabolizes NE. Both H_2_O_2_ and DOPEGAL induce δ-secretase activation and trigger cell death, and we have shown that the transcription factor C/EBPβ regulates the expression of δ-secretase^50^. Conceivably, there is positive feedback between NE metabolism and C/EBPβ, whereby increased levels of MAO-A and H_2_O_2_ activate C/EBPβ, which, in turn, induces further MAO-A and δ-secretase expression.

## DOPEGAL triggers AEP activation, Tau N368 cleavage, and cytotoxicity

A previous study showed that DOPAL covalently modifies α-synuclein and promotes its aggregation^31^. Interestingly, DOPEGAL also directly modifies Tau and induces its aggregation in a concentration-dependent manner, which has been validated by the accumulation of high-molecular-weight bands. As expected, δ-secretase-truncated Tau N368 recombinant proteins are more prone to aggregation than Tau FL. DOPEGAL strongly augments Tau fibrillization over time, as revealed by the high-intensity ThT fluorescence of Tau preformed fibrils (PFFs), while NE and DOPAL inhibit Tau fibrillization^68^. This finding suggests that DOPEGAL presumably covalently modifies Tau on active lysine or cysteine residues, escalating its aggregation. In alignment with the in vitro results, DOPEGAL provokes Tau aggregation in noradrenergic-like SH-SY5Y cells transfected with human Tau. DOPEGAL (but not DA or NE) produces demonstrable Tau aggregation, correlating with its hyperphosphorylated (AT8-positive) status. Moreover, DOPEGAL also upregulates total δ-secretase levels and its proteolytic activation, as well as the abundance of Tau N368, and induced SH-SY5Y cell death. Importantly, although δ-secretase is similarly activated by DOPEGAL in both WT and Tau^−/−^ neurons, its toxicity was significantly attenuated in Tau-deficient neurons, suggesting that Tau is necessary for the full extent of DOPEGAL-induced cell death. WT Tau promotes cell death, which is exacerbated by the Tau N368 fragment. Remarkably, Tau N368 displays neurotoxicity comparable to Tau P301S, whereas prevention of δ-secretase cleavage blunts Tau P301S toxicity, indicating that δ-secretase cleavage contributes to Tau neurotoxicity, fitting with our previous finding^5^. Accordingly, overexpression of MAO-A stimulates Tau-mediated cell death via δ-secretase cleavage of Tau N368. Dopamine β-hydroxylase (DBH) is required for NE synthesis. Consequently, DBH depletion reduces δ-secretase activity, diminishes Tau N368 cleavage, and Tau-triggered cell death^68^. Furthermore, overexpression of either MAO-A or MAO-B substantially provokes δ-secretase activation, and blockade of these enzymes by small molecular inhibitors attenuates δ-secretase enzymatic activity, Tau hyperphosphorylation, and N368 cleavage. Therefore, these data support the following model: MAO-A oxidizes NE into DOPEGAL and elicits oxidative stress, triggering δ-secretase activation and Tau N368 cleavage and cytotoxicity. On the other hand, DOPEGAL is also metabolized in vivo via reduction by aldehyde reductase (AR) and further oxidation by aldehyde dehydrogenase (ALDH). Accumulation of DOPEGAL via blockade of its metabolism with the AR inhibitor imirestat, the ALDH inhibitor daidzein, or both drugs augments its cytotoxicity. Imirestat and the mixture of both inhibitors escalate δ-secretase activation, Tau N368 cleavage, and AT8 abundance in combination with MAO-A overexpression^68^. Again, inhibition of DOPEGAL catabolism results in its accumulation, further supporting our model.

## DOPEGAL escalates δ-secretase-cleaved Tau N368, Tau pathology and LC degeneration in a Tau and δ-secretase-dependent manner

Immunofluorescence (IF) staining in the LC of postmortem human AD brains and different ages of transgenic AD mouse models shows that Tau N368 cleavage correlates with Tau hyperphosphorylation. AT8 and Tau N368 immunoreactivity are absent from WT mouse brains but they appear and accumulate together in a time-dependent manner in the LC of both 3×Tg mice and Tau P301S mice. Compared with healthy controls, LC sections from AD subjects exhibit the same effect, indicating that δ-secretase is temporally activated in the LC, cleaving Tau N368 and triggering its hyperphosphorylation. AT8/N368-positive Tau is also ThS-positive in the LC region of 12-month-old 3×Tg mice, 6-month-old Tau P301S mice, and human AD subjects, supporting that hyperphosphorylated Tau is aggregated, and Tau fibrillization is further confirmed by Gallyas-Braak staining. As expected, activated δ-secretase is abundant in the diseased LC, where it is associated with Tau N368, hyperphosphorylated Tau, and aggregation^68^.

DBH^+/−^ littermates possess normal NE levels, and DBH knockout (DBH^−/−^) mice lack NE completely (and thus cannot produce DOPEGAL)^69^. Although the abundance of pathological Tau escalates in both genotypes at 6 to 9 months, AT8, Tau N368, and Gallyas-Braak silver staining are significantly reduced in Tau P301S/DBH^−/−^ mice compared with Tau P301S/DBH^+/−^ mice, supporting that DOPEGAL is implicated in Tau pathology and LC neuronal degeneration. Consistent with the finding that suppression of NE production protects against Tau toxicity in vitro, TH staining reveals a loss of LC neurons in Tau P301S/DBH^+/−^ mice at 9 months that is partially abrogated by DBH^−/−^. Consequently, Morris water maze (MWM) cognitive-behavioral tests show that comparable age-dependent deficits are observed at 3 and 6 months in NE-deficient and NE-competent Tau P301S mice. However, at 9 months, the cognitive performance of Tau P301S/DBH^−/−^ mice was significantly better than that of Tau P301S/DBH^+/−^ mice. Because DOPEGAL promotes the activation of δ-secretase, DBH^−/−^ mice that cannot produce DOPEGAL are protected from Tau pathology. As expected, Tau P301S overexpression elicits much greater Tau pathology than the uncleavable Tau P301S N255A/368 A mutant. Strikingly, the ability of Tau P301S to trigger AT8, Tau N368, and Gallyas-Braak staining is attenuated significantly in DBH^−/−^ mice, suggesting that NE promotes Tau P301S N368 cleavage by δ-secretase and its subsequent phosphorylation and aggregation.

**Fig. 4:**
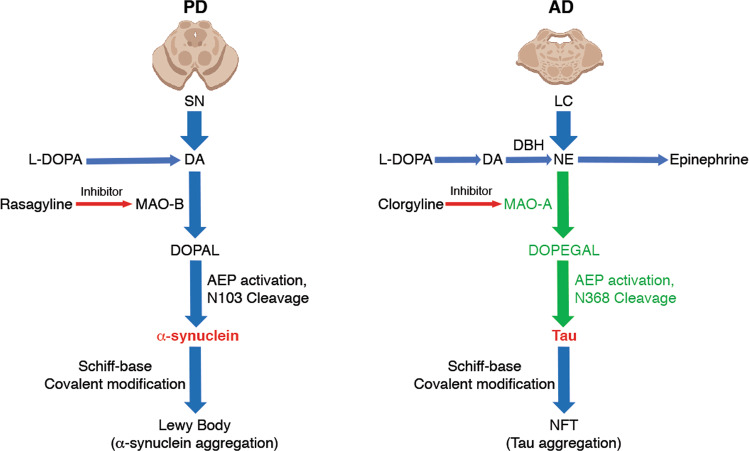
Schematic models for the selective vulnerability of dopaminergic and noradrenergic neurons in Parkinson’s disease and Alzheimer’s disease. DA metabolites by MAO-B and DOPAL display toxic effects in PD pathology specifically in the substantia nigra, leading to δ-secretase activation and alpha-synuclein cleavage and toxicity (left). Similarly, MAO-A and its toxic NE metabolite, DOPEGAL, induce tau truncation and toxicity by activating δ-secretase in noradrenergic cells of the locus coeruleus in the early stage of AD progression (right).

MAO-A and DOPEGAL are upregulated in the LC of postmortem AD brains^61^, and DOPEGAL (but not NE or its other oxidative or O-methylated metabolites) is toxic to differentiated PC12 cells^60^. Accordingly, administration of DOPEGAL into the LC of 2-month-old WT, Tau^−/−^, MAPT transgenic mice that overexpress WT human Tau, and MAPT/AEP^−/−^ mice elicits greater LC neuronal apoptosis in MAPT mice than WT mice, and these effects are abrogated by Tau or δ-secretase knockout, indicating that DOPEGAL-induced degeneration of LC neurons in vivo is partially mediated by a Tau- and δ-secretase-dependent mechanism. In alignment with these findings, acceleration of endogenous NE metabolism and DOPEGAL production by overexpressing MAO-A displays similar effects. As a result, MAO-A triggers significant cognitive dysfunction in MAPT mice compared with control virus, whereas it has no effect on WT mice. Hence, Tau exacerbates DOPEGAL-mediated LC neuronal toxicity and cognitive impairment^68^. The close neurochemical relationship between NE and DA suggests that the toxicity of the DA metabolite, DOPAL, may have a correlate in the noradrenergic system. Physiological concentrations of DOPAL trigger the formation of α-synuclein oligomers and aggregates in both a cell-free system and in cell culture and produce cytotoxicity in vitro and in vivo. We recently reported that DOPAL activates δ-secretase in dopaminergic neurons and leads to α-synuclein N103 cleavage, resulting in its aggregation and dopaminergic neuronal degeneration in the SN^58^. Thus, DOPAL-α-synuclein interactions may underlie the selective vulnerability of DA neurons in PD^30,31^ (Fig. 4, left panel). Accordingly, a similar interaction might occur between DOPEGAL and Tau in AD. Conceivably, DOPEGAL may covalently modify the lysine and cysteine residues and oxidize Met groups on Tau, thus facilitating its conformational change and fibrillization. Imaginably, MAO-A mediates the conversion of NE into DOPEGAL, which induces Tau toxicity in the LC and triggers the selective vulnerability of LC neurons in AD (Fig. 4, right panel).

## Delta-secretase cleavage of Tau in LC initiates Tau pathology and mediates its spreading

Hyperphosphorylated Tau appears exclusively in the LC in Braak stage 0, spreads to the entorhinal cortex (Cx) in Braak stages I and II, and then propagates to the hippocampus and frontal Cx in stages III–VI^17^. Because the LC is the first brain structure to develop Tau lesions and has widespread connections to other areas of the brain and Tau is capable of transsynaptic propagation, LC neurons have been proposed as the critical initiators of the stereotypical spread of Tau pathology in AD^19,21^. Consistent with this notion, injection of AAV-mCherry + AAV-Tau under control of the noradrenergic-specific PRSx8 promoter into the LC^70^ of 3-month-old MAPT mice elicits robust AT8 and N368 Tau immune reactivity in the LC neurons. Noticeably, AT8 aberrant Tau spreads from the LC to the cerebellum, midbrain, hippocampus, entorhinal Cx, and Cx. Moreover, δ-secretase-cleaved Tau N368 and aggregated Tau are present in the entorhinal Cx (EC), hippocampus (HC), and Cx, indicating that Tau pathology spreads from the LC to these distal regions. Cognitive-behavioral tests demonstrate that Tau-injected mice spend significantly less time in the target quadrant during the MWM probe trial and are impaired in both cued and contextual freezing following fear conditioning. Thus, LC-derived Tau pathology can spread to the forebrain and produce cognitive impairment^68^. Tau N368 is tightly associated with AT8 immunoreactivity, suggesting that δ-secretase cleavage of Tau may facilitate its spread. This finding is corroborated with primary LC neurons from neonatal WT and AEP^−/−^ mice and AAV-PRSx8-hTau, and human Tau is truncated at N368 and hyperphosphorylated in WT LC neurons, whereas both N368 and AT8 immunoreactivity are attenuated in AEP^−/−^ LC neurons. In alignment with this observation, AT8, N368 Tau, and Gallyas-Braak staining indicate that hyperphosphorylated and δ-secretase-cleaved Tau accumulation in the LC and subsequent spread to the HC, Cx, and EC are largely retarded in AEP^−/−^ mice. Moreover, deletion of δ-secretase prevents cognitive impairment induced by Tau overexpression in the LC. Therefore, these data indicate that δ-secretase contributes to the spread of LC-derived Tau pathology to the forebrain and associated cognitive deficits^68^.

Our study shows that Tau pathology originating in the LC is capable of propagating to interconnected brain regions, including those that also show early vulnerability in AD. Remarkably, both the spread of Tau pathology and the accelerated cognitive deficits following Tau expression in the LC were alleviated in AEP^−/−^ mice^68^. Accumulating studies have now documented Tau aggregate uptake, “seeding” (i.e., aggregate serving as a template for the conversion of monomer to fibrillar aggregates) and transfer of aggregates among cultured cells. Tau fibril propagation between connected neurons with seeding of Tau monomer in recipient cells mediates this progression in vivo^71^. Importantly, injection of Tau aggregates into mice that express human Tau induces Tau pathology that spreads outward along known brain networks^72^. Therefore, propagation of an aggregated state underlies the progression of Tau pathology. Tau forms discrete prion “strains” that propagate with remarkable fidelity through living systems^73^. Injection of homogenate from different tauopathy brains into a mouse model that expresses full-length human Tau induces pathology that closely resembles that of human source cases^74^. Interestingly, Lee and her colleagues report that Tau fibrils purified from AD brains (AD-Tau), but not synthetic Tau fibrils, result in the formation of abundant Tau inclusions in anatomically connected brain regions in nontransgenic mice. Recombinant human Tau seeded by AD-Tau reveals unique conformational features that are distinct from synthetic Tau fibrils, which could underlie the differential potency in seeding physiological levels of Tau to aggregate^75^. Moreover, inoculation of Tau P301S mice or nontransgenic mice with different pathological Tau strains causes strain-specific intracellular pathology in distinct cell types and brain regions and induces different rates of network propagation^76^.

Neuroinflammation is an important feature in the initiation and progression of AD pathologies. Microglia, which are primary innate immune cells in the brain, play an important role in the propagation of Tau protein as well as coordinating the inflammatory processes in AD progression. Microglia propagate Tau protein via exosome secretion, and the inhibition of exosome synthesis suppresses Tau propagation^77^. AEP may be related to microglia-induced Tau propagation, since AEP knockout alleviates spreading Tau pathology and AEP is abundantly expressed in microglia. Furthermore, a recent study demonstrated that Tau cleavage by AEP is a physiological event in activated microglial cells in AD brains^78^.

We recently reported the examination of AD patients’ CSF Tau levels using both commercial and novel assays in relation to [^18^F]THK5317 (Tau) and [^18^F]FDG PET (glucose metabolism). The changes in the levels of Tau N368 CSF markers track the longitudinal changes in tracer uptake better than changes in P-tau_181p_ and T-Tau levels and improve concordance with dichotomized regional [^18^F]THK5317 measures in AD patients^79^. Moreover, the levels of all forms of CSF Tau are inversely associated with baseline [^18^F]FDG uptake. The Tau N368/t-Tau ratio was significantly decreased in AD (*P* < 0.001) in all cohorts. IHC staining demonstrates that Tau N368 is present in tangles. There is a strong negative correlation between the CSF Tau N368/t-Tau ratio and ^18^F-GTP1 retention. Hence, Tau N368 is a tangle-enriched fragment, and the CSF ratio Tau N368/t-Tau reflects tangle pathology. This novel Tau biomarker could be used to improve the diagnosis of AD and to facilitate the development of drug candidates targeting Tau pathology^80^. Most recently, we show that δ-secretase cleaves α-synuclein at N103 and Tau at N368 and mediates their fibrillization and retrograde propagation from the gut to the brain, triggering nigra dopaminergic neuronal loss associated with Lewy bodies and motor dysfunction. α-Synuclein N103 and Tau N368 robustly interact with each other and are highly elevated in the gut and brain of PD patients. Preformed fibrils (PFFs) of α-synuclein N103/Tau N368 are more neurotoxic and compact and aggregate more quickly along the vagus nerve than their FL/FL counterparts or the individual fragments’ fibrils. Colonic injection of PFFs induces PD pathologies, motor dysfunctions, and cognitive impairments. Thus, δ-secretase plays a crucial role in initiating PD pathology progression from the ENS to the CNS^81^. Mounting evidence supports that α-synuclein and Lewy bodies are also implicated in AD brains. A fragment of α-synuclein, called the non-Aβ component (NAC), was originally identified in samples containing AD senile plaques^82^. There is a growing body of evidence that Aβ enhances α-synucleinopathy. For instance, a postmortem study suggests that the existence of AD pathology accelerates Lewy pathology and the progression of DLB^83^. Aβ-enhanced α-synucleinopathy was further supported by the finding of enhanced aggregation and accumulation of α-synuclein by Aβ^84^, as well as accelerated accumulation of p-α-synuclein in double transgenic mice with mutant APP and presenilin-1^85^. Conceivably, δ-secretase-truncated α-synuclein N103 and Tau N368 aggregates may also contribute to AD pathogenesis.

## Tau pathology exacerbates AD pathologies in the forebrain

The amyloid cascade hypothesis has dominated the AD field for more than two decades, and it states that the accumulation of Aβ in the brain is the primary influence driving AD pathogenesis. The rest of the disease process, including the formation of NFT, is proposed to result from an imbalance between Aβ production and Aβ clearance^86^. This hypothesis posits that the deposition of Aβ in the brain is the cause of neural/synaptic damage and dementia. The supportive evidence for this hypothesis includes the following: (1) all dominant mutations causing early-onset AD occur either in the substrate (APP) or the protease (presenilin) of the reaction that generates Aβ^87^. Duplication of the wild‐type APP gene in Down’s syndrome leads to Aβ deposits in teens^88^. Apolipoprotein E4 (APOE4), which predisposes patients to AD in >40% of cases, has been found to impair Aβ clearance from the brain^89^. Human Aβ oligomers also induce hyperphosphorylation of Tau at AD‐relevant epitopes and cause neuritic dystrophy in cultured neurons^90^. However, mounting evidence challenges this oversimplified one-way amyloid-Tau degenerative cascade. For example, many cognitively normal elderly subjects have relatively large amounts of Aβ in their brains^91^. Moreover, Aβ PET studies in cognitively normal subjects showed that almost one-third of elderly individuals have major amounts of Aβ in their brains^92^. Most importantly, clinical trials with therapeutics that aim to reduce the levels of amyloid-β in the brain have failed^93^, which leads to questions of the role of Aβ in AD. In addition, in double transgenic mice expressing both human mutant Tau and APP, greater Aβ deposition and NFT-like formation with increased neuronal loss are found when compared with APP or Tau single transgenic mice, indicating that Tau may accelerate amyloid deposition^94^. Interestingly, human secreted Tau increases Aβ production in human neurons^95^. Therefore, these conflicting findings indicate that the causal link between aberrant APP processing and Tau alteration remains controversial. Most recently, we show that δ-secretase-truncated Tau N368 elicits STAT1 nuclear translocation and enhances BACE1 expression, leading to Aβ production escalation. Consequently, the fragments of δ-secretase cleavage, APP (586-695), and Tau (1-368), additively drive AD pathogenesis and cognitive dysfunctions. Thus, Tau may not only be a downstream effector of Aβ in the amyloid hypothesis; it may also act as an upstream driving force for Aβ when cleaved by δ-secretase^40^. These findings are consistent with previous observations that intraneuronal Tau alterations precede aggregated Aβ deposition in Braak stage I-III in the presymptomatic stages of AD, and Tau tangles develop temporally either before or independent of Aβ plaques^96^.

In humans, the apolipoprotein E (ApoE) gene has three polymorphic alleles (ε2, ε3, and ε4), with the ε3 allele being the most common (77%) and ε2 allele the least common (8%). The ε4 allele increases the risk for both familial and sporadic AD^97^, and up to 60% of people with AD carry at least one ε4 allele, compared to only 15% of healthy controls. ApoE is an apolipoprotein abundant in cholesterol- and triglyceride-rich plasma lipoproteins and is essential for Aβ deposition in APP transgenic mice. Notably, Aβ clearance is slower in ApoE4-TR mice than in ApoE3-TR mice^98^. Although a large number of clinical and preclinical studies suggest that ApoE isoforms impact AD pathogenesis by driving Aβ pathology, ApoE4 also impacts neurodegeneration in the context of Tau pathology, independent of Aβ. The presence of ApoE4 exacerbates Tau-mediated neurodegeneration, whereas the absence of ApoE is neuroprotective^99^. Injection of Tau preformed filaments (PFFs) or Tau virus into the LC leads to progressive loss of LC neurons and the appearance of Tau pathology in the forebrain^68,72^. Presumably, ApoE and Tau may crosstalk with each other in the LC, mediating AD pathologies. NE is mainly sequestered in the VMAT2 vesicles, and its MAO-A-oxidized metabolite DOPEGAL is predominantly generated in the cytoplasm, where NE may be leaked^100^. Conceivably, DOPEGAL may activate δ-secretase, triggering Tau N368 fragmentation and subsequent aggregation, which may spread to the forebrain via transsynaptic propagation of NFT-like Tau pathology to anatomically connected brain regions, e.g., LC projections into the hippocampus.

## Concluding remarks

Truncated and hyperphosphorylated Tau is prone to aggregation, and this “pretangle” form of the protein appears in the locus coeruleus (LC), the major central source of norepinephrine (NE), before any other area of the brain, making it the earliest detectable AD-like neuropathology. Hyperphosphorylated Tau appears exclusively in the LC in Braak stage 0 (a-c), spreads to the entorhinal cortex (EC) in Braak stages I–II, and then propagates to the hippocampus and frontal cortex in stages III–VI. Interestingly, we show that DOPEGAL, which is produced exclusively in noradrenergic neurons by MAO-A metabolism of norepinephrine, activates δ-secretase that cleaves Tau at residue N368 into aggregation- and propagation-prone forms, thus leading to LC degeneration and the spread of Tau pathology. In the forebrain, aggregated Tau N368 may crosstalk with Aβ and ApoE4, promoting AD pathology onset. Collectively, our studies lay a solid foundation that δ-secretase is a crucial drug target that drives AD pathogenesis.

Using high-throughput screening, we obtained the promising lead compound #11 that selectively blocks δ-secretase and is brain permeable. Based on the cocrystal structures between the lead compound and active δ-secretase, we can optimize δ-secretase inhibitors into a preclinical candidate for treating AD. Hopefully, inhibition of δ-secretase will simultaneously block APP and Tau proteolytic cleavage, mitigate Abeta and Tau pathology and decrease neurodegeneration, alleviating the cognitive deficits in AD patients.
